# Promising targets of cell death signaling of NR2B receptor subunit in stroke pathogenesis

**DOI:** 10.1186/2050-490X-2-8

**Published:** 2014-07-23

**Authors:** Shu Shu, Lei Pei, Youming Lu

**Affiliations:** Department of Pathophysiology, Tongji Medical College and Institute for Brain Research, Huazhong University of Science and Technology, 13# Hangkong Road, Wuhan, 430030 PR China

**Keywords:** Stroke, DAPK1, NMDA receptor, Neuronal death

## Abstract

Stroke is an acute cerebrovascular disease caused by acute brain artery bursting or cerebral embolism that leads to neuronal death and severe dysfunction of synaptic transmission. Neuronal damage after stroke remains a major cause of morbidity and mortality worldwide and affects 795 000 of lives every year in United States. However, effective treatments remain lacking, which makes the identification of new therapeutic targets a matter of great importance.

N-methyl-D-aspartate glutamate (NMDA) receptor is important both in the normal synaptic transmission and in the neuronal death after stroke. Accumulated evidences show NMDA receptor downstream effectors, such as PSD-95, DAPK1, and ERK, had been revealed to be linked with neuronal damage. Based on our recent studies, we review the promising targets of the NMDA receptor downstream signaling involved in stroke treatment. This review will provide the concept of NR2B downstream signaling in neuronal death after stroke and provide evidences for developing better NMDAR-based therapeutics by targeting downstream proteins.

## Review

Stroke is one of the most life- threatening cerebrovascular disorders, the second leading cause of lethality and major cause of disability in the world. In consequence of interruption of cerebral blood flow, Stroke causes irreversible damage to the affected neurons. There are two main types of stroke, ischemic and hemorrhagic. Ischemic stroke accounts nearly for 85% of all reported stroke incidents. This type of stroke occurs when a thrombus or embolus blocks cerebral blood flow resulting in cerebral ischemia and consequent neuronal damage and cell death. Hemorrhagic stroke, accounts for the remaining 15% stroke cases, occurs due to rupture of blood vessel in the brain which produces rapid cerebral injury. Intravenous recombinant tissue plasminogen activator (rtPA) is the only FDA approved drug for treatment of ischemic stroke [1]. Patients who receive this drug within the therapeutic window (<4.5 hours) also have a high risk of intracranial bleeding, usually 6–8% against 0.6–2% spontaneous hemorrhages in stroke. Other limitations associated with rtPA therapy like disruption of blood brain barrier, seizures and progression of neuronal damage are major concerns. Thus, there is a stringent need for exploring novel neuroprotective strategies for the treatment of ischemic stroke [2].

Recent studies on the blocking peptides of NR2B downstream cell death signaling pathway have revealed their potential neuroprotective roles in ischemic stroke. The cell death blocking peptides have shown promising effects on protecting the neurons against excitotoxic insults, and on decreasing infarct volume and improving neurological functions in experimental models of ischemic stroke [3]. This review briefly focuses on the promising targets involved in the downstream cell death signaling of NR2B receptor subunit and their potential in the treatment of cerebral ischemic stroke.

### NR2B downstream signaling in stroke

The N-methyl-D-aspartate receptor (also known as the NMDA receptor or NMDAR), a glutamate receptor, is the predominant molecular switch for controlling synaptic plasticity and memory function [4]. Excessive stimulation of the NMDAR is an initial and crucial event for neuronal death after cerebral ischemic stroke [5]. It is known that synaptic NMDA receptor NR2A subunits play an important role in calcium ions (Ca_2_+) permeation of cell physiological reaction [6], while extrasynaptic NR2B subunit receptor links signal transmission of cell death. In particular, the mechanisms that control the recruitment of cell death or cell survival pathways upon activation of NMDARs are thought to depend in part, on the Ca^2+^ concentration and its route of entry, but mostly on the subunit composition and localization of the NMDARs that it activates [7]. Several evidences have suggested that heteromeric NR1/NR2B receptors are initial triggers of cell death pathways, while NR1/NR2A receptors mediates cell survival signaling. First, in both mature cortical cultures and in animals in vivo, the activation of NR2B-containing NMDARs results in excitotoxicity, while the activation of NR2A-containing NMDARs promotes neuroprotection [8]. Second, NR2B-containing NMDARs are localized preferentially at extrasynaptic sites while NR2A-containing NMDARs are expressed at the synaptic area [9], and activation of extrasynaptic NMDARs and associated downstream signaling cascades correlates with a pro-death transcriptional response while activation of synaptic NMDARs lead to pro-survival transcriptional response [10]. Third, neurotoxicity induced by glutamate release from astrocytes involves extrasynaptic NR2B-containing NMDARs [11]. Finally, glutamate sensitivity in neurons increases in parallel with the expression level of NR1/NR2B as NR2B-containing NMDARs have a higher affinity for glutamate, slower deactivation kinetics, and reduced Ca^2+^ -dependent desensitization when compared to NR2A-containing receptors [12].

Hence blocking NR2B subunit is thought to be the best target to block ischemic injury [13]. However, NR2B subunit has been found to combine with NR1/NR2A subunit and to form NR1/NR2A/NR2B receptor assembly at synapses [14]. A large family of synthetic compounds that selectively inhibit NMDARs containing the NR2B subunit [15]. Among them, several highly potent molecules show good efficacy as neuroprotectants in a variety of animal models. It is noteworthy that in humans, NR2B-selective antagonists do not induce the adverse side effects usually seen with nonselective NMDAR antagonists, even at maximally neuroprotective doses [16]. Despite these encouraging data, NR2B-selective antagonists have not succeeded in clinical trials yet because of uncertain biosafty and pharmacokinetic profiles [17]. Consequently, the selectivity of NR2B subunit antagonist acting on extrasynaptic NMDA receptor is facing challenges. New potent NR2B-selective antagonists are still in great demand [18].

Overactivation of NMDAR induces calcium overload, oxidative/nitrosative stress and excitotoxicity in neuronal cells. These are considered as the major pathogenesis of cerebral ischemic stroke [19–21]. Excitotoxicity is one of the essential theories of cell death in stroke (including ischemic stroke and hemorrhagic stroke) [22]. It’s generally recognized that glutamate, a kind of excitatory neurotransmitters, excessively accumulates in ischemic tissue to activate postsynaptic glutamate receptors, including α-amino-3-hydroxy-5-methyl-4-isoxazole-propionic acid receptor (AMPA receptor) and NMDA receptor at ischemic and anoxic state. Especially, NMDA receptor allows a mass of Ca^2+^ influx, leading to Ca^2+^ overload [22] and activating a series of signaling molecules. Calcium overload causes mitochondria damage, generation of reactive oxygen species (ROS) and reactive nitrogen species (RNS), and then leads to cell necrosis and autophagy [23, 24]. These ROS and RNS have two effects, one is inhibiting the signal transduction of cells regulating in oxidation; the other is directly inducing necrosis [25]. In stroke injury, it is known that main sources of ROS include the mitochondria electron transport chain and the oxidative phosphorylation of NADPH-oxidase. Hence, NADPH-oxidase is thought to be a potential target for stroke treatment. Accumulated facts prove that oxidative stress is the major inducement of stroke damage. Since oxidative stress participates in chronic inflammation and metabolic disorders [26]. Astrocytes, neuronal support cells, contribute to inflammation during stroke insult. Astrocytes, under normal conditions, perform physiological functions like, glutamate uptake, glutamate release and maintain cellular and ion homeostasis [27]. During cerebral injury, astrocytes undergo morphological changes and become activated. Activated astrocytes release proinflammtory cytokines and chemokines thus results in initiation and progression of inflammation [28]. In cerebral ischemia, microglia, residential brain macrophages, become activated and release detrimental neurotoxic mediators like proinflammtory cytokines, superoxide, nitric oxide (NO), TNF- α and proteases [29]. Many of these mediators can in turn influence the microglia morphology and activate it in a paracrine and autocrine fashion. The inhibition of this pathway of neuronal damage can be considered as a logical option. NO is a small molecule, secreted by microglia cells, acts directly on neurons as neurotoxin or indirectly by potentiating excitotoxic transmitters. Inhibition of microglia activation in ischaemia may provide a novel target in management of stroke [30].

Researchers have been working on clearing ROS to block this vicious circle and hope for fighting stroke for years. However, lots of clinical trials show that clearing ROS does not help for preventing the progress of stroke damage, but even worsens it in some situation [31]. Because ROS has important physiological function in maintaining tissue homeostasis, clearing ROS will damage the normal functions of the central nervous system, such as balancing the electrolyte balance, stabling the osmotic pressure and so on [32]. Therefore, we hold the opinion that stroke can be treated by limiting the source of oxidative stress through selectively enhance the capability of endogenous antioxidant system without impairing the normal functions of ROS in the brain. In addition, mitochondrial abnormality can induce DNA damage through activating apoptotic signaling pathway, and then induce cell apoptosis at late stage.

**Figure 1:**
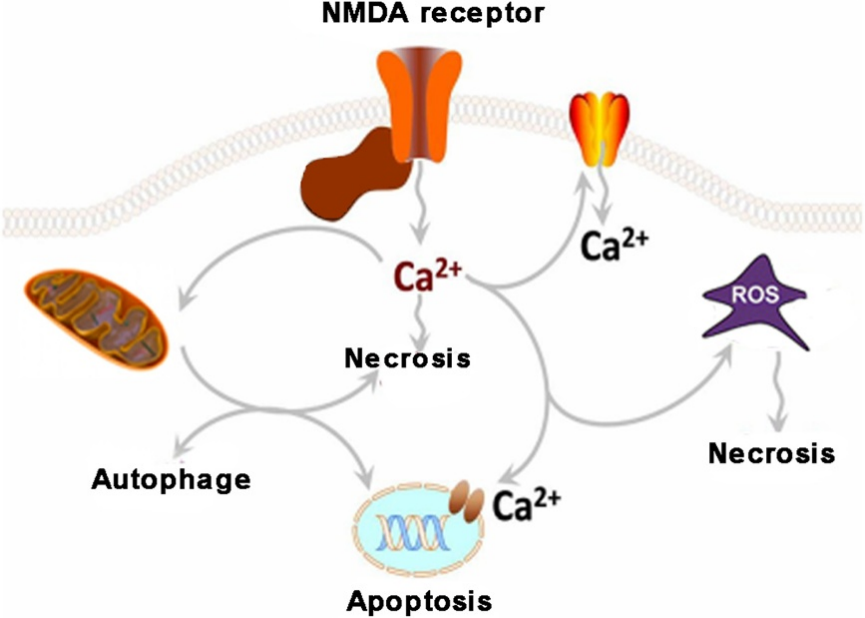
**Excitatory neurotransmitter (glutamate) acts at extrasynapses and over-activates NMDA receptors in stroke.** Over-activation of NMDA receptor leads to excessive Ca^2+^ loading into cells. Overloading of Ca^2+^ induce mitochondria injury, thus causes necrosis and generation of ROS/RNS. On the other hand, excessive intracellular Ca^2+^ also activate Ca^2+^ channels on nuclear membrane, causing DNA damage and cell apoptosis. DNA damage also promotes the expression of apoptosis and autophagy related genes, and finally induce cell apoptosis and autophagy.

Although activation of glutamate receptor is the major cause of cell death (including necrosis, apoptosis and autophagy) in stroke (Figure 1), almost all clinical strategies which attempt to block NMDA receptors are failed in stroke treatment [33]. The main reason is that blocking NMDA receptors also interrupts physiological function of synaptic transmission. Therefore, we supposed that explore the specific downstream substrates of NMDA receptor and selectively block cell death signaling pathway without affecting physiological functions might be the ideal way on stroke therapy.

### Downstream signals of NR2B receptor subunit in stroke

NR2B subunit is a modular structure consisting of four distinct domains: an extracellular N-terminal domain, an extracellular ligand binding domain, a transmembrane domain and an intracellular C-terminal domain of variable length [34, 35]. The transmembrane domain, containing three membrane-spanning helices and a membrane re-entrant loop, contributes to the channel pore formation. The cytoplasmic domain is required for protein-protein interactions. This interaction with the intracellular domain of NMDAR subunits is subject to extensive posttranslational modifications such as phosphorylation, which is essential to regulate downstream signaling [36]. The carboxyl terminus of NMDA subunit binds important intracellular signaling complexes, allowing for their efficient and selective activation by calcium influx through the opening of NMDAR channels [37]. Therefore, selective inhibition of NMDAR mediated neuronal injuries may be achieved.

#### NR2B-DAPK1 signaling

**Figure 2:**
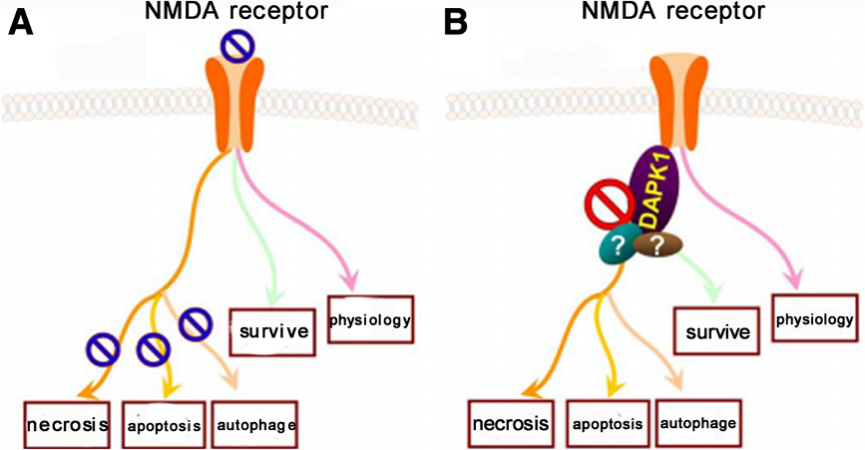
**Blockade of NMDA receptor or NMDA-DAPK1 downstream signaling induces different fates of neuronal cells in stroke. (A)** Blockade of NMDA receptor in stroke causes 1) inhibition of necrosis, apoptosis and autophagy of neuronal cells; 2) suppression of endogenous surviving and regeneration signaling; 3) interruption of physiological functions of synaptic transmission. **(B)** Blockade of NMDA(NR2B)-DAPK1 downstream signaling in stroke specifically mediates 1) prevention of cell death (such as necrosis, apoptosis and autophagy); 2) probable inhibition of cell survival; and 3) no adverse effects on physiological functions of synaptic transmission.

Recently, a new signaling molecule engaged in the neuronal death cascade was identified by using coimmunoprecipitation with NR2B-specific antibodies and mass spectrometry. It is found that after stroke injury, NMDAR interacts with Death Associated Protein Kinase 1 (DAPK1) through NR2B subunit, a member of a serine/threonine kinase family well known for their roles in cell death [38]. Stroke injury activates DAPK1, which migrates to extrasynaptic site and binds to NR2B receptor subunit. Through GST-pull down experiments combined with coimmunoprecipitation, our colleagures demonstrate that DAPK1 directly interacts with the NR2B via an interaction in the amino acids fragment from 1297 to 1304 in C-terminal of NR2B subunit (NR2Bi) specifically. By defining the exact binding domain of DAPK1 in NR2B, an interference peptide (NR2B_CT_) was designed to effectively disrupt the interaction of DAPK1 with NMDARs during stroke. It is worth noting that NR2B_CT_ could alleviate infarction area and improve neurological score after ischemic stroke. In short, the combination of DAPK1-NR2B receptor subunit mediates pathological process of stroke injury, including necrosis, apoptosis and autophagy of neuronal cells (Figure 2), and most importantly, it is not relevant to physiological function of NMDA receptor because the interrupting of DAPK1 with NR2B does not alter the synaptic transmission of NMDA receptor [39]. Our recent work further demonstrated that the signaling events downstream of DAPK1 involve the interaction of DAPK1 with p53 (a tumor suppressor) and the followed activation of proapototic transcription-dependent and -independent pathways. Through disruption of the DAPK1–p53 interaction by a small membrane-permeable peptide inhibitor (Tat-p53DM) effectively blocked these two distinct pathways of neuronal death in ischemia [40]. Thus, DAPK1 can be considered as a signaling amplifier of downstream signaling of NR2B subunits at extrasynaptic sites for mediating brain damage in stroke, and targeting NR2B-DAPK1-p53 signaling can be considered as a practical strategy for stroke therapy.

#### NR2B-PSD95-nNOS signaling

Interaction of NMDA receptor NR2B subunit and postsynaptic density protein-95 (PSD-95) has been firstly demonstrated on focal cerebral ischemic injury by using the yeast two-hybrid screen technique [41]. Furthermore, PSD-95 acts as a scaffolding protein links the NR2B carboxyl terminus to the death-signaling protein nNOS. nNOS is an enzyme that catalyzes the production of highly neurotoxic molecule nitric oxide (NO) [42].

Studies have shown that disruption of the NR2B-PSD-95-nNOS signaling complex inhibits NMDAR-mediated NO release and prevents neuronal death. For example, Tat-NR2B9c, that competes with native NR2B subunits for binding to PSD-95 and dissociates PSD-95-nNOS from NR2B, could systemically reduce ischemic brain damage in rat model of stroke [43]. In addition, Zhou and his colleagues further examined the essential role of this NMDAR signaling complex in ischemic neuronal damage. By co- immunoprecipitation, the authors found that the interaction between PSD-95 and nNOS is greatly enhanced in the brain after stroke. Interestingly, the increase in PSD-95–nNOS interaction in neurons after stroke requires both activation of NR2Bsubunit containing NMDARs (NR2BRs) and active association of NR2B with PSD-95 [44]. Interestingly, by competing with nNOS for binding to PSD-95, the nNOS-N_1–133_ peptide effectively limited the interaction between PSD-95-nNOS and reduced the brain damage after stroke [45, 46]. These results add to the growing evidence that NR2B–PSD-95–nNOS interaction is required for ischemic brain damage mediated by the NMDAR [47]. Nevertheless, as NMDA stimulation can induce nNOS activation at doses lower than that required to induce neurotoxicity, and such NMDAR-mediated nitric oxide production may be involved in brain functions such as synaptic plasticity required for learning and memory [48, 49]. Thus, future studies are needed to solve these questions.

#### NR2B-ERK signaling

ERK1/2 signaling pathway, is activated by Ca^2+^ influx through NMDARs, plays an important role in NMDAR-dependent neuronal survival. Extrasynaptic NMDAR activity inactivates ERK signaling pathway, while activation of synaptic NMDARs induces sustained ERK activation [50, 51]. Consistent with being more abundant at extrasynaptic sites, NR2B has been shown to be selectively associated with synaptic Ras GTPase activating protein (SynGAP) [52], which represses ERK signaling. In addition, suppression of NMDA-dependent ERK activation by the dominant-negative form of RasGRF1 suggests that RasGRF1 is a crucial intermediate between the NMDAR and ERK activation. RasGRF1 is a Ras-specific GDP/GTP exchange factor (GEF) and Ras activator. RasGRF1 is selectively expressed in the central nervous system (CNS) and is enriched in synapses. RasGRF1 activates Ras only when it binds Ca^2+^/calmodulin and is thus a potential target of Ca^2+^ entering neurons [53]. The interaction between NR2B and RasGRF1, and the demonstration that disruption of this interaction attenuated NMDA-dependent ERK activation, strongly suggests that the NR2B subunit directly links the NMDAR to ERK activation [54].

As is known, stimulation of either synaptic or extrasynaptic NMDARs is linked to the activation of ERK/mitogen-activated protein kinases (MAPK), which therefore appear to play a dual role as prosurvival [55] and prodeath kinases [56]. Another interesting finding is that tau toxicity is accompanied by sustained and delayed activation of ERKs and that this activation is NMDAR dependent and critically involved in cell death. Tau protein supports the microtubule system responsible for intracellular transport, axonal morphology, and cell physiology. The correct functioning of tau depends upon a balance between the different tau isoforms, its state of phosphorylation, and its structural integrity. A perturbation of these parameters may cause tau dysfunction and tau toxicity [57]. More importantly, tau influences NMDAR signaling and induces a necrotic type cell death [13] is demonstrated by (i) the rapidity of neurotoxic effect, (ii) the lack of classical hallmarks of apoptosis, (iii) the neuroprotective role offered by an inhibitor of extrasynaptic NR2B receptor, and (iv) the dephosphorylation of CREB. Furthermore, two MEK inhibitors, U0126 and PD98059, as well as genetic ablation of ERK1, inhibit tau toxicity, in agreement with previous report that ERKs exert critical role in oxidative glutamate toxicity in cortical neurons and in other paradigms of neuronal death [58, 59].

So far, there is no direct evidences show the roles of NR2B-ERK signaling in ischemic stroke models. However, the above research results may throw light on this potential signaling pathway mediating neuronal cell death, and provide clues that interrupt NR2B-ERK pathway may be another target for preventing neuronal death after stroke. Therefore, next studies should focus on this area to address this issue.

## Conclusion

In conclusion, directing at NMDA receptor, many stroke protecting drugs (219 drugs were tested in clinical trials around the world) have been developed and used to fight against stroke. However, none of these drugs have achieved success. Because all these drugs severely interrupt the physiological functions of NMDA receptor and leading to serious side effects such as cognitive disorder, hallucination, dyspnea and even coma [20]. In this review, we summarized several experimental stroke studies and identified specific components of NMDARs downstream signaling complexes responsible for neuronal death. These reports have led to the recent development of several therapeutic peptides that exert neuroprotective actions by specifically disrupting the downstream neuronal death signaling pathways of NMDARs without affecting the physiological functions.
